# Significantly increased risk of chronic obstructive pulmonary disease amongst adults with predominantly mild congenital heart disease

**DOI:** 10.1038/s41598-022-21433-8

**Published:** 2022-11-04

**Authors:** Dominic J. F. Byrne, Simon G. Williams, Apostol Nakev, Simon Frain, Stephanie L. Baross, Jørgen Vestbo, Bernard D. Keavney, David Talavera

**Affiliations:** 1 Division of Cardiovascular Sciences, School of Medical Sciences, The University of Manchester, Oxford Road, Manchester, UK; 2 Cybersecurity Department, Institute for Infocomm Research, Agency for Science, Technology and Research, Singapore, Singapore; 3 Division of Infection, Immunity & Respiratory Medicine, School of Biological Sciences, The University of Manchester, Oxford Road, Manchester, UK; 4 Manchester Heart Institute, Manchester University NHS Foundation Trust, Manchester, UK

**Keywords:** Computational biology and bioinformatics, Congenital heart defects, Chronic obstructive pulmonary disease

## Abstract

Adults with congenital heart disease (CHD) face increased risk of various comorbid diseases. Previous work on lung dysfunction in this population has mainly focused on restrictive lung disease, in patients with severe CHD phenotypes. We examined the association of mild CHD with chronic obstructive pulmonary disease (COPD) in the UK Biobank (UKB). Electronic health records (EHR) were used to identify 3385 CHD cases and 479,765 healthy controls in UKB, before performing a case–control analysis over a 20-year study period for a total of > 9.5 M person-years of follow-up. Our analysis showed that UKB participants with CHD are at substantially greater risk of developing COPD than healthy controls (8.7% vs 3.1% prevalence, unadjusted OR 2.98, 95% CI 2.63, 3.36, P = 1.40e−53). Slightly increased rates of smoking were observed amongst CHD cases, however the association with COPD was shown to be robust to adjustment for smoking and other factors known to modulate COPD risk within a multivariable-adjusted Cox regression framework (fully adjusted HR 2.21, 95% CI 1.97, 2.48, P = 5.5e−41). Care for adults with CHD should aim to mitigate their increased risk of COPD, possibly via increased smoking cessation support.

## Introduction

Congenital heart disease (CHD) is the most common developmental defect amongst live births; its recorded birth prevalence globally is increasing^1,2^. Significant advances in clinical management over recent decades mean that adults with CHD now outnumber affected infants^3^. The growth of this adult population has demanded investigation into the burden of comorbidity associated with CHD in adulthood, such that improvements in understanding can inform lifelong care.

Such work has identified comorbid diseases that are more prevalent among adults with CHD, including various forms of cardiovascular disease^4^ and chronic renal disease^5,6^. Although recent studies have suggested that the burden of chronic obstructive pulmonary disease (COPD) amongst adults with CHD may be greater than previously thought^6,7^, no sizeable prospective study with the capacity to adjust for important environmental factors predisposing to COPD has yet been conducted. Moreover, although isolated bicuspid aortic valve (BAV) represents the commonest cardiovascular malformation, it is under-represented within the spectrum of congenital heart disease studies, and data on pulmonary comorbidity in BAV is lacking. We investigated the risk of COPD diagnosis amongst adults with CHD in the UK Biobank (UKB) cohort, among whom it is known most CHD is mild, and BAV predominates^4,8^.

## Results

### Characteristics of the study population

We identified 3385 participants with CHD, of which 1960, 1294 and 131 were sub-classified as having isolated aortic valve, noncomplex and complex defects, respectively. The complex CHD group was deemed too small for meaningful subgroup analyses and thus it was not separately analysed, however these cases were retained in the main CHD group. The CHD group overall was observed to contain a greater proportion of males (57.5% vs 45.2%, P = 4.94e−46). We anticipated this overrepresentation of male participants since 58% of the CHD diagnoses were attributed to bicuspid aortic valve, which is typically reported to be around three times commoner in males than females. Table 1 shows the characteristics of the case and control groups. See Table S1 for a similar breakdown of the characteristics of the two major CHD subgroups.

**Table 1:** Characteristics of the study cohort.

	All CHD		Control		P-value
	Male	Female	Male	Female	
n	1945 (57.5)	1440 (42.5)	216,791 (45.2)	262,974 (54.8)	–
Age, median (IQR)	60 (53, 64)	58 (52, 63)	58 (50, 63)	57 (50, 63)	2.04E−18
White, n (%)	1859 (95.6)	1374 (95.4)	205,288 (94.7)	248,940 (94.7)	0.03
TDI, median (IQR)	− 1.86 (− 3.44, 1.11)	− 1.84 (− 3.39, 1.02)	− 2.15 (− 3.66, 0.58)	− 2.16 (− 3.65, 0.44)	1.99E−10
BMI, median (IQR), kg/m^2^	27.8 (25.4, 30.9)	27.2 (24.0, 31.2)	27.3 (25.0, 30.0)	26.1 (23.4, 29.7)	1.22E−28
**Smoking status, n (%)**					
Current	238 (12.2)	139 (9.7)	27,154 (12.5)	23,419 (8.9)	0.26
Former	856 (44.0)	486 (33.8)	82,877 (38.2)	82,535 (31.4)	4.72E−10
Never	851 (43.8)	815 (56.6)	106,760 (49.2)	157,020 (59.7)	2.13E−11
Pack years of smoking, median (IQR)	25.0 (13.0, 40.4)	19.0 (9.8, 33.9)	21.0 (11.0, 35.0)	16.5 (8.5, 28.1)	6.27E−14
**Other diagnoses, n (%)**					
Asthma	278 (14.3)	281 (19.5)	27,077 (12.5)	37,328 (14.2)	3.19E−07
Hypertension	1,502 (77.2)	941 (65.3)	133,843 (61.7)	128,858 (49.0)	1.5E−95

P-values refer to differences between participants of both sexes in the CHD and control groups. Median/IQR for pack years of smoking were calculated using only values from ever-smokers. *TDI* Townsend deprivation index.

Participants of both sexes with CHD were observed to have a greater burden of socioeconomic and behavioural factors commonly associated with increased COPD risk when compared to controls. These include age at recruitment (median age in years Males: 60 vs 58, Females: 58 vs 57, P = 2.04e−18), self-described White ethnicity (Males: 95.6% vs 94.7%, Females: 95.4% vs 94.7%, P = 0.03), material deprivation (median Townsend deprivation index Males: −1.86 vs −2.15, Females: −1.84 vs −2.16, P = 1.99e−10), proportion of current/former smokers (Males: 56.2% vs 50.8%, Females: 43.4% vs 40.3%, P = 2.13e−11) and pack years of smoking amongst ever smokers (median pack years Males: 25.0 vs 21.0, Females: 19.0 vs 16.5, P = 6.27e−14). Pack year data was only available for ~ 66% of ever-smokers in UKB. The CHD group displayed greater rates of asthma (Males: 14.3% vs 12.5%, Females: 19.5% vs 14.2%, P = 3.19e−7) and hypertension (Males: 77.2% vs 61.7%, Females: 65.3% vs 49.0%, P = 1.50e−95). Cases also had higher BMI compared to controls (median kg/m^2^ Males: 27.8 vs 27.3, Females: 27.2 vs 26.1, P = 1.22e−28), which may be protective against COPD-related mortality^9^. Significant interaction effects were observed between sex and CHD for age at recruitment (P = 7.61e−3), BMI (P = 6.97e−3) and diagnoses of asthma (P = 0.02). Similar relationships were observed for both the noncomplex and isolated aortic valve (AoV) groups independently, except for the noncomplex group showing no significant difference in age at recruitment or percentage White ethnicity when compared to controls.

### COPD diagnosis in CHD patients and controls

**Figure 1:**
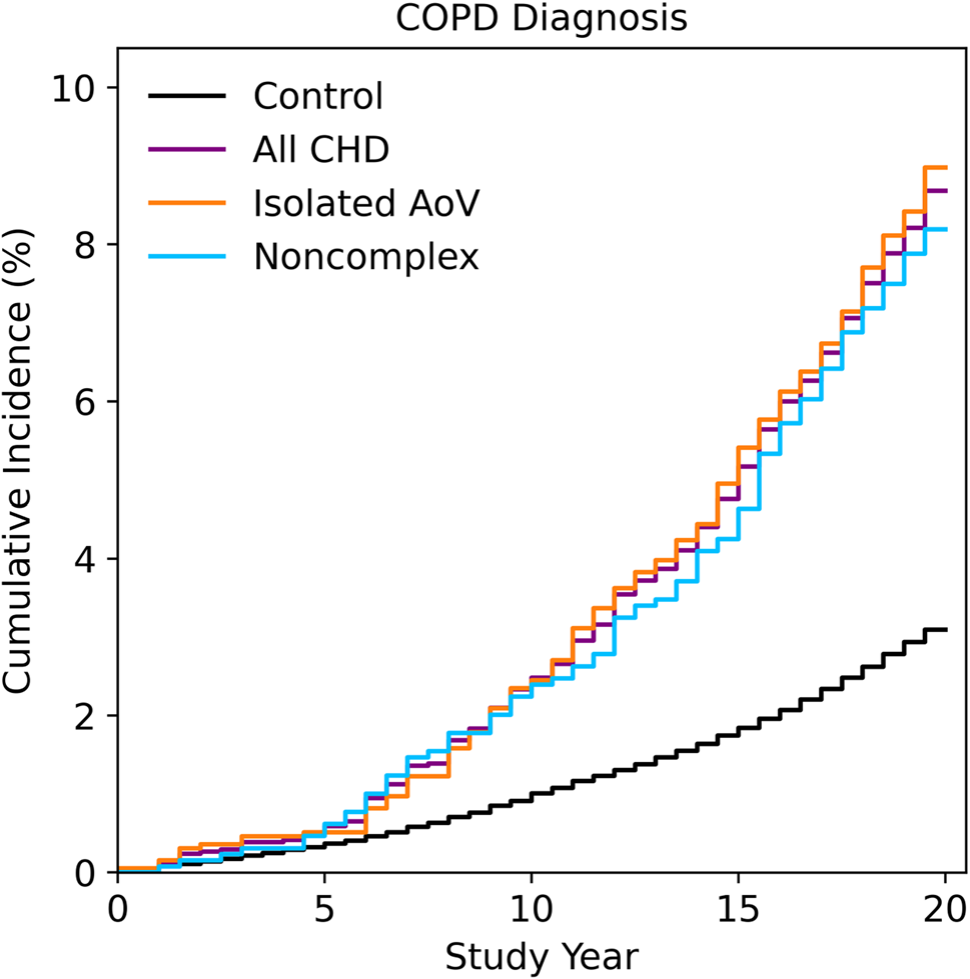
Cumulative incidence of COPD diagnosis amongst the CHD and control groups, along with the two major CHD subgroups. These data represent 20 years of follow up from the study start date (01/04/1997) to the end of available records (31/03/2017).

We observed greater unadjusted rates of COPD diagnosis amongst the CHD group as a whole (294 diagnoses, OR 2.98, 95% CI 2.63, 3.36, P = 1.40e−53), in the isolated AoV subgroup (176 diagnoses, OR 3.09, 95% CI 2.63, 3.61, P = 1.02e−34) and in the noncomplex subgroup (106 diagnoses, OR 2.79, 95% CI 2.27, 3.41, P = 8.72e−19) when compared to healthy controls (14,854 diagnoses) over the 20 year study period (Fig. 1). The number at risk in each group at each five-year time point is presented in Table S2.

COPD diagnosis remained associated with CHD following adjustment for a range of factors known to modulate COPD risk. Importantly, adjustment for age and sex, which were imbalanced between the CHD cases and controls, had only a small effect on the hazard ratios for COPD, which remained substantial and highly significant (Table 2). Adjustment for other COPD risk modulators, including smoking status (current vs former vs never), ethnicity (White vs non-White), BMI, material deprivation (Townsend deprivation index), and diagnoses of hypertension and asthma did not materially attenuate the association. We noted that after full adjustment, the hazard ratios in the Isolated AoV group (which had an approximately two-thirds male preponderance) and in the Noncomplex group (which had approximately equal numbers of males and females) were of similar magnitudes and had similar levels of statistical support.

**Table 2:** Hazard ratios (HR) for COPD diagnosis across CHD groups.

	All CHD	Isolated AoV	Noncomplex	Control
No. at risk	3385	1960	1294	479,765
COPD diagnoses, n (%)	294 (8.7)	176 (9.0)	106 (8.2)	14,854 (3.1)
**Age/sex adjusted**				
HR	2.63	2.54	2.73	–
95% CI	(2.34, 2.95)	(2.19, 2.94)	(2.25, 3.30)	–
P-value	2.69E−60	1.46E−34	7.18E−25	–
**Fully adjusted**				
HR	2.21	2.11	2.39	–
95% CI	(1.97, 2.48)	(1.82, 2.45)	(1.97, 2.89)	–
P-value	5.50E−41	6.93E−23	4.57E−19	–

Similar results to those shown in Table 2 were observed after fitting an alternate model, in which the smoking status variable was replaced with a categorical “pack years of smoking” variable (never smoker vs 0–10 vs 10–20 vs 20–30 vs > 30 pack years), though numbers were smaller as these data are available on fewer UKB participants (Table 3).

**Table 3:** Fully adjusted hazard ratios (HRs) for COPD diagnosis across all CHD groups.

	All CHD	Isolated AoV	Noncomplex	Control
No. at risk	2854	1633	1107	409,222
COPD diagnoses, n (%)	257 (9.0)	153 (9.4)	92 (8.3)	13,122 (3.2)
HR	2.04	1.89	2.34	–
95% CI	(1.80, 2.31)	(1.61, 2.21)	(1.90, 2.87)	–
P-value	1.30E−29	7.01E−15	5.58E−16	–

These HR estimates are adjusted for smoking via inclusion of a categorical pack years of smoking (Never smokers, 0–10, 10–20, 20–30, > 30) predictor. Aside from the method of adjusting for smoking (and exclusion of participants without pack year data) this model is identical to the main fully adjusted model present in Table 2. No. at risk refers to the number of participants in each group for which all relevant data were available.

### Sensitivity analyses

We carried out a sensitivity analysis to assess the potential that unintended inclusion of participants with non-congenital aortic valve defects into the case cohort had affected our results. We fitted an additional model after lowering the age limit for inclusion based on diagnosis/treatment of aortic valve disease from 65 to 50 years. This resulted in exclusion of ~ 2/3rds of the isolated AoV group (as expected, given that this was the major inclusion stream for suspected BAV cases), but did not materially affect the results of the analysis (Table 4).

**Table 4:** Estimated hazard ratios (HR) for COPD diagnosis across all CHD groups after adjustment for both age/sex and full adjustment, which incorporates the same predictors as those found in the main Cox regression model whose results are presented in Table 2.

	All CHD	Isolated AoV	Noncomplex	Control
No. at risk	2055	673	1251	479,765
COPD diagnoses, n (%)	158 (7.7)	43 (6.4)	103 (8.2)	14,854 (3.1)
**Age/sex adjusted:**				
HR	2.58	2.05	2.82	–
95% CI	(2.20, 3.01)	(1.52, 2.76)	(2.32, 3.42)	–
P-value	2.52E−32	2.69E−06	1.21E−25	–
**Fully adjusted:**				
HR	2.25	1.89	2.46	–
95% CI	(1.92, 2.63)	(1.40, 2.55)	(2.02, 2.98)	–
P-value	4.74E−24	3.15E−05	1.14E−19	–

These results were obtained after lowering the age limit for inclusion based on diagnosis/treatment of aortic valve disease from 65 to 50 years old. For a full list of the relevant diagnosis codes, see Table S6. 43 patients self-reported having surgery on an unspecified heart valve between the ages of 50 and 65 and were removed from the noncomplex case group in view of diagnostic uncertainty regarding whether this had been an aortic valve operation.

Whilst diabetes mellitus is an established comorbidity of COPD, it is typically not thought to play a causative role in the aetiology of obstructive lung disease^10^. However, in light of some evidence to the contrary^11–13^, we conducted a sensitivity analysis to assess the possibility that the decision not to adjust for diagnoses of diabetes could have impacted the results of our analysis. We found that inclusion of diabetes as a predictor in an additional fully-adjusted Cox regression model brought about no significant change in the estimates of the COPD HR for either the main CHD group or the two major subgroups (Table S3).

We analysed spirometry data collected at recruitment to assess the risk that our findings were due to an unmeasured reporting bias brought about by increased clinical supervision of the adult CHD population, compared to healthy controls. As a cross-sectional measure, any effect observed in these data would be independent of any reporting biases. A reduction in the ratio between pre-bronchodilator measurements of forced expiratory volume in 1 s (FEV_1_) and forced vital capacity (FVC) (FEV_1_/FVC) was observed in CHD cases compared to controls, which was robust to adjustment for known modulators via multivariable linear regression (Table S4). Further details are provided in the Supplementary Materials.

Finally, we performed a sensitivity analysis to investigate whether the definition of COPD used in this study-based on electronic health records (EHR) and self-reported illnesses/operations- could have led to errant results. Due to the absence of the adequate spirometric data we were unable to assess if all COPD diagnoses were correct or whether there were instances of misidentification. An alternate binary outcome phenotype was defined based on (1) presence of an EHR of self-reported diagnosis of COPD prior UKB recruitment, along with (2) airflow obstruction during spirometric assessment at recruitment. CHD was strongly associated with this outcome after adjustment for known modulators via multivariable logistic regression (Table S5). Further details are provided in the Supplementary Materials.

## Discussion

Our results demonstrate that adults with predominantly milder forms of CHD are at substantially greater risk of developing COPD when compared to healthy controls. Analysis of the two major CHD subgroups, participants with isolated AoV defects (mostly consisting of BAV cases) and those with noncomplex defects (chiefly atrial/ventricular septal defects and other non-cyanotic malformations—see Table S6H,I for a full description), showed that the increased burden of COPD is relatively uniform across different types of mild CHD. These associations are robust to adjustment for various behavioural and socioeconomic factors which are known to modulate COPD risk. Therefore, despite observing enrichment of known risk factors for COPD amongst adults with CHD, the higher rate of COPD diagnosis in this population did not appear to be due to confounding by these factors.

Previous studies indicate that the majority of adults with CHD have no history of lung disease, but have shown a high prevalence of abnormal spirometry, in particular restrictive defects. The pattern of lower FVC has been particularly noted in tetralogy of Fallot and in patients with a Fontan circulation, suggesting that reduced pulmonary blood flow in utero and during early life importantly influences this predisposition^14^. In a study of 1188 adults with CHD who had spirometry data, Alonso-Gonzalez et al. showed 47.2% had a restrictive pattern (characterised by FVC < 70% predicted and FEV_1_/FVC > 0.7) while only 5% had a pure obstructive pattern^14^. Neidenbach et al. reported non-cardiac comorbidities in 800 adults with inherited and congenital heart disease from a single centre, of whom 20% were coded as having simple lesions by the ACC classification^15^. Lung disease was observed in 16% of their cohort. Singh et al. studied non-cardiac comorbidities in ACHD patients hospitalised in the US during 2013–2014 and found a much larger proportion (20%) of these hospitalised cases to have COPD than other previous studies, or the present study^7^. However, there was no case–control comparison in Singh et al.’s study and it was not designed to estimate the prevalence of COPD in a representative (i.e. largely non-hospitalised) CHD population. Bracher et al. studied comorbidities in a cohort of 1725 CHD cases from a single centre, reporting a 6.7% prevalence of either obstructive or restrictive lung disease, which was not further subclassified^16^. Yang et al. carried out a case–control comparison of comorbidities in 2122 CHD patients and 8488 controls to determine mediators of any relationship between CHD and depression^6^. This study, alone among previous investigations, did find an increased relative risk of COPD in CHD cases (OR 2.08, 95% CI 1.75, 2.47), but the database utilised did not contain smoking data, so no adjustment for the main COPD risk factor could be made. Nevertheless, it is of interest that the point estimate for that previous study lies within the 95% confidence interval of our fully-adjusted HR for COPD among CHD patients compared to controls (Table 2). These previous studies into abnormalities of lung function have chiefly recruited patients with moderate or severe CHD phenotypes; the novelty of the present study arises from the fact that it analyses the biggest cohort of mild CHD defects. This allows for subgroup analyses, including the largest number of cases of BAV reported in a single dataset thus far, and a subset of noncomplex CHD that is comparable in size to previous investigation.

In light of our findings, the increased rate of smoking history in the CHD group when compared to sex-matched controls is concerning. While current smoking was not significantly different between CHD cases and controls, there were fewer never smokers among the CHD cases. Our findings in the UKB study are at variance with some previous studies of this question. Engelfriet et al. observed lower smoking frequency amongst 3375 adults with CHD^17^, although the difference from our observations could be due in part to the comparatively younger and more severe cases of CHD in their study. Zomer et al. also observed reduced smoking frequency amongst adults with CHD, even in cases of mild severity^18^. Regardless, in the context of the significant burden of COPD we describe, and the previously reported increased atherosclerotic morbidity in the UKB population with CHD^4^, further research to investigate this issue is warranted. If confirmed in other cohorts, our findings would suggest that more intensive monitoring of lung function, and counselling on the risks of smoking, may be appropriate in the adult CHD clinic, even for those with mild CHD.

Whilst these results clearly demonstrate that adults with mild CHD are at greater risk of developing COPD, the mechanism underlying this disease association remains unclear. Given that the overwhelming majority of CHD cases in this cohort have mild cardiac defects, the possibility that the increased risk of COPD in this population is due to aberrant cardiac output is highly unlikely. One recent study identified aortopathies in neonates with BAV, suggesting that BAV-associated aortopathy is a congenital malformation^19^. This establishes that the developmental mechanisms responsible for BAV can affect the elastic behaviour of structures beyond the aortic valve. This same mechanism could similarly affect the elastic structures of the airways and alveoli, which may contribute to the increased risk of COPD in this population. However, we accept that there is no direct evidence for this hypothesis and further work is needed to elucidate the mechanism underlying this disease association.

A limitation of this study is the use of a purely anatomical classification system for defining subgroups of CHD cases, rather than a system which also incorporates physiology such as the ACHD/AP system^20^. Unfortunately, the EHR and self-reported illness/operations data used to identify CHD cases in this cohort does not contain sufficient granularity to properly use such a classification system. However, whilst the ACHD/AP system has been shown to be more performant in predicting mortality^21^ we argue that benefit in particular is of limited relevance to this study, which is focused on comorbid associations. As such, we contend it is reasonable to use a purely anatomical classification system, as has been done in this cohort by others^4^.

One potential source of bias is that the increased rates of COPD diagnosis we observed amongst CHD cases arose in part due to reporting biases brought about by the increased levels of attention clinicians may pay to the respiratory health of adults with CHD, compared to healthy controls. However, the observation that the CHD group had lower adjusted pre-bronchodilator FEV_1_/FVC ratios, both before and after excluding participants with previous diagnoses for asthma or COPD (Table S5), stands in contrast to this idea. Nonetheless, respiratory health screening of adults with mild CHD, such as those in this study, is likely to be either minimal or non-existent in most cases. As such, any reporting bias is unlikely to be the main driver of this disease association, particularly given the magnitude of the effect we report.

Another consideration is that increased rates of cardiopulmonary bypass surgery, which is known to lead to poorer respiratory health outcomes^22,23^ in CHD cases may have been responsible for their greater burden of COPD diagnosis. Major surgeries such as these to correct severe cardiac defects would have almost exclusively taken place during early life. Unfortunately, EHR data covering this period was not available in UKB, preventing further investigation of this potential source of bias. Regardless, we would expect such surgeries to be rare in our cohort, given the mild types of CHD we observed. In particular, BAV cases would likely not have undergone any corrective surgeries at all and the increased burden of COPD in the isolated AoV group (in which BAV predominates) remains substantial.

In summary, we have shown a novel comorbid association between mild CHD and COPD in the UK Biobank cohort. Together with recent observations demonstrating a higher risk of coronary artery disease in CHD patients in UK Biobank^4^, these findings indicate that CHD patients are at significantly higher risk of two largely preventable later-life diseases. Our findings could inform a greater emphasis on the risks posed to CHD patients by these comorbid conditions, in routine clinical care.

## Methods

Informed consent for all participants was collected by UKB during the recruitment process. UKB was approved by the North West Multi-Centre Research Ethical Committee (REF: 11/NW/03820) and all research was performed in line with the relevant ethical guidelines. This study was performed in line with the principles of the Declaration of Helsinki.

### CHD phenotype classification

**Figure 2:**
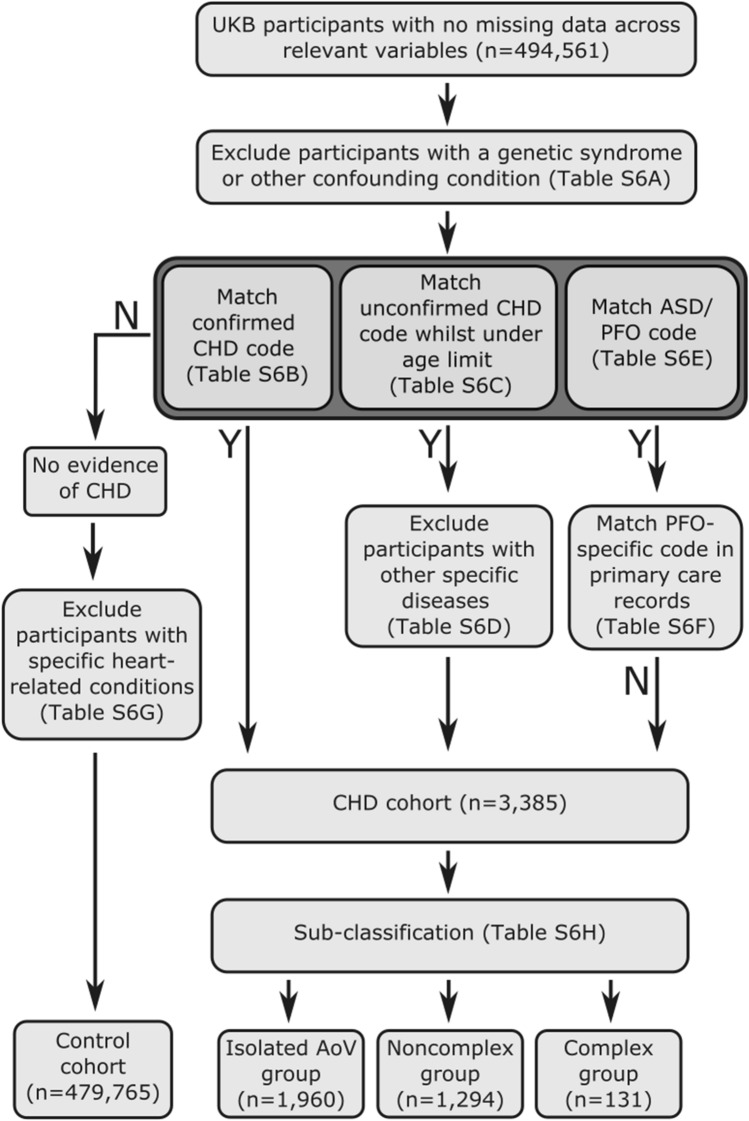
Classification scheme used to define case and control cohorts for non-syndromic CHD in UKB.

The study design of UKB and details of participant recruitment have been previously described^24^. Among the 500,000 study participants, CHD was defined in this study as any structural malformation of the heart or great vessels, inclusive of BAV, in the absence of a pleiotropic genetic syndrome. Definition of CHD case and control cohorts was achieved via a multi-step classification scheme (Fig. 2), similar to our previous studies^8,25^ and work by others^4^.

The classification of UKB participants utilised three main sources of data: hospital episode statistics (HES), primary care records and illnesses/procedures self-reported by participants during recruitment to UKB. HES data provides information on participant hospital admissions, with illnesses detailed using ICD-9 and ICD-10 codes and procedures using OPCS-4 codes. Primary care records detail visits made by participants to their respective GPs, with both diagnoses and procedures encoded using CTV3 codes. Primary care records were available for ~ 45% of UKB participants at the time of study.

Participants with codes indicating presence of a genetic syndrome or a potentially confounding cardiac condition (Table S6A) were excluded from both case and control cohorts. Participants with codes clearly indicating the presence of a heart defect with confirmed congenital aetiology (Table S6B) were included into the case cohort. Next, participants with codes indicating the presence of a structural heart defect of unconfirmed aetiology (Table S6C) were included as cases, provided they did not also have any codes indicating that the heart defect in question was of non-congenital origin (Table S6D).

Inclusion into the case cohort on the basis of codes indicating the presence of an aortic valve (AoV) defect was limited to participants aged 65 or younger at the time of diagnosis. This threshold was used to distinguish participants with congenital AoV defects (primarily BAV) from those with age-related degeneration of a developmentally normal (i.e., tricuspid) aortic valve. Further details are available in the Supplementary Materials, and a sensitivity analysis examining the validity of using this age cutoff can be found in the Results section.

The final inclusion stream aimed to identify participants with an atrial septal defect (ASD). Critically, the ICD-10 code for ASD (Q21.1) can also be used for patent foramen ovale (PFO). As PFO is present in up to 25% of the population, is clinically insignificant in the overwhelming majority of cases, and is not typically considered a CHD phenotype, we implemented a further filter to remove cases from the CHD group whose ASD could not be reliably distinguished from PFO. Further details are available in the Supplementary Materials.

Participants included in the CHD group were further classified into three subgroups, comprising complex, noncomplex and isolated AoV defects, based on a similar classification to that used by others working on this cohort^4^. Further details can be found in Table S5H and I. Participants with complex CHD were retained in the main CHD group but excluded from subgroup analyses due to the small number of cases identified.

### Classification of respiratory phenotypes

**Figure 3:**
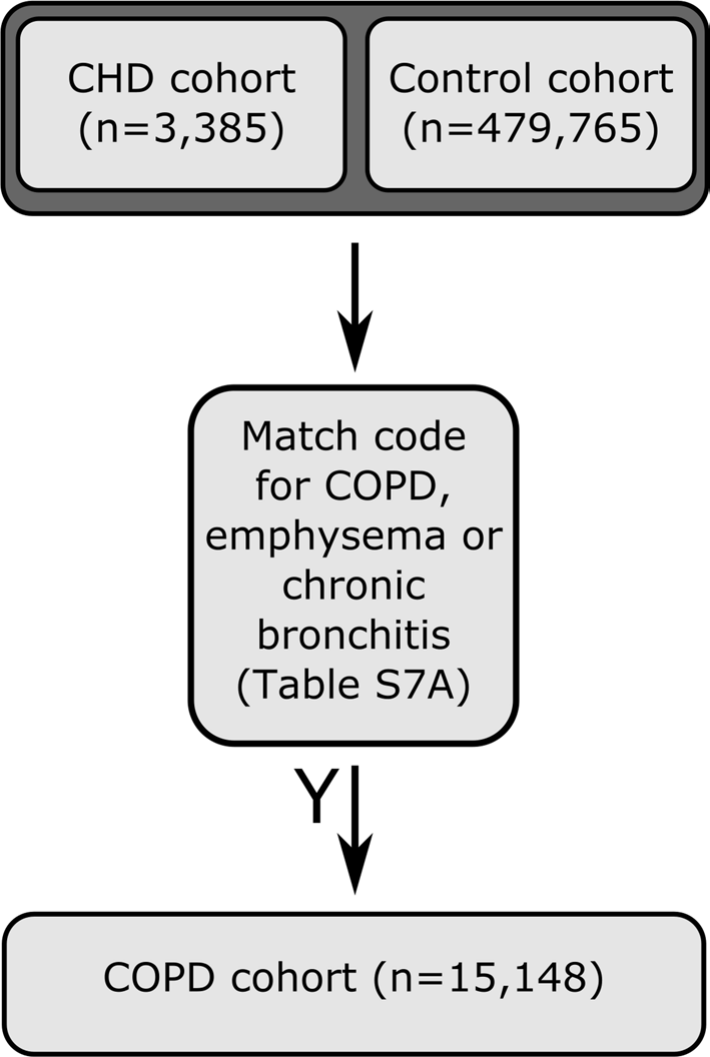
Classification scheme used to identify COPD cases in UKB.

We classified chronic obstructive pulmonary disease (COPD) based on presence of EHR (inclusive of primary care, hospital inpatient and death records) and self-reported illness codes (Table S7A) which either directly indicated COPD, or indicated emphysema or chronic bronchitis (Fig. 3). COPD classification was limited to codes present in patients records from 01/04/1997, as this was the earliest date from which HES records were available for all the devolved nations of the UK.

Classification of participants as asthmatic or having a history of pulmonary tuberculosis (TB) was based on presence of relevant EHR/self-reported illness codes (Table S7B and C, respectively). We opted not to classify participants as asthmatic on the basis of self-reported bronchodilator use, since these medications are also prescribed in the treatment of COPD.

Pre-bronchodilator measurements of FEV_1_ and FVC were made at the UKB assessment centre (AC). The ratio between these measures is a key diagnostic criterion for airflow obstruction (FEV_1_/FVC). These data were available on 268,549 study participants, after removing those not meeting ERA/ATS reproducibility standards^26^.

### Classification of hypertension

Classification of participants as hypertensive was based on presence of relevant EHR/self-reported illness codes (Table S7D). In addition, participants who self-reported taking blood pressure medication, or who were measured to have either diastolic blood pressure > 90 mmHg or systolic blood pressure > 140 mmHg at the UKB AC, were also classified as hypertensive.

### Classification of diabetes mellitus

Classification of participants as diabetic was based on presence of relevant EHR/self-reported illness codes (Table S7E). In addition, participants who self-reported use of insulin were also classified as diabetic.

### Statistical analyses

Differences in categorical variables across participant groups were tested for significance using Fisher’s exact test. Comparisons of continuous variables across groups were tested for significance using the Mann–Whitney *U* test. Tests of interaction between CHD and sex for continuous and binary variables were performed using linear and logistic regression, respectively.

Hazard ratios (HRs), along with relevant 95% confidence intervals and P-values, were estimated using multivariable Cox proportional hazard regression analyses, conducted with the lifelines package in Python^27^. Adherence to the proportional hazards assumption for each of the Cox models was assessed using the Schoenfeld residuals test. In the main regression models on COPD diagnosis, this test indicated that the following variables did not meet this underlying model assumption: diagnosis for asthma or hypertension, smoking status and Townsend deprivation index. To account for this, the relevant models were stratified on the problematic variables, which allows HRs of interest to be properly adjusted without requiring that the subgroups defined by each variable share baseline hazard functions, therefore preserving the requirement for proportional baseline hazards for each of the model predictors. Participants were first placed into groups based on 5 equal-width bins of Townsend deprivation index values to enable stratification, given the continuous nature of this variable. Participants with any missing data for any of the variables included in a given regression model were excluded from the analysis.

Whilst pulmonary TB infection is a risk factor for COPD, we chose not to include it as a predictor in the main regression analyses of COPD diagnosis. Further discussion of the rationale behind this decision and a sensitivity analysis (Table S8) are included in the Supplementary Materials.

## Supplementary Information

Supplementary Information.

## Data Availability

UK Biobank is an open-access resource. Bona fide researchers can apply to use the UK Biobank dataset by registering and applying at http://ukbiobank.ac.uk/register-apply/. Code used in these analyses has been made freely available by the authors at https://github.com/dombyrne/SciRep_Byrne_2022.
