# Supplementary material for: Significantly increased risk of chronic obstructive pulmonary disease amongst adults with predominantly mild congenital heart disease
Source: Sci Rep. 2022 Nov 4;12:18703. doi: 10.1038/s41598-022-21433-8 (PMC9636374; doi:10.1038/s41598-022-21433-8)
Supplement: Supplementary file 1 — Supplementary Information. [file 41598_2022_21433_MOESM1_ESM.docx]

# Supplementary Materials

## Contents

Supplementary results

Supplementary methods

References

Tables S1-S7

# Supplementary results

## Spirometric comparison of airflow limitation and vital capacity in CHD cases and controls

Pre-bronchodilator FEV_1_/FVC ratio, measured at the UKB AC, was significantly lower amongst the CHD group than controls after adjustment for sex and age at recruitment (Table S4). Subsequent adjustment for material deprivation (Townsend deprivation index), BMI and smoking status (current vs former vs never), as well as age and sex, made little difference to the estimated effect size. Removal of all participants who had a diagnosis of asthma or COPD prior to their spirometric measurement at the first UKB AC had limited impact on these results. Adjustment for ethnicity was not required in these models, as all the UKB participants with all relevant data available uniformly self-described as White ethnicity. The authors recognise that the use of pre-bronchodilator measurements of FEV_1_ represents a limitation to this study, however post-bronchodilator measures were not available.

## Analysis of airflow obstruction amongst CHD cases and controls at recruitment using a hybrid outcome phenotype

An alternate binary outcome phenotype was defined based on presence of an EHR/self-reported COPD diagnosis prior to the date of UKB recruitment along with a pre-bronchodilator FEV_1_/FVC ratio of <0.7 as measured at recruitment. This outcome was enriched amongst CHD cases (1.5% vs 0.7% prevalence) but did not reach significance prior to adjustment (P: 0.06), possibly due to the limited number of COPD diagnoses which occurred before recruitment to UKB. CHD was significantly associated with the outcome following adjustment for age and sex only, as well as full adjustment for age, sex and the array of modulators of COPD risk included in the other regression analyses in this study, within a multivariable logistic regression framework (Table S5). As with other analyses using spirometry data, the authors recognise that the use of pre-bronchodilator measurements of FEV_1_ represents a limitation of this study, however post-bronchodilator measures were not available.

# Supplementary methods

## Classification of aortic valve defects

Inclusion into the case cohort on the basis of codes indicating the presence of an aortic valve (AoV) defect was limited to participants aged 65 or younger at the time of diagnosis. This threshold was used to distinguish participants with congenital AoV defects (primarily BAV) from those with age-related degeneration of a developmentally normal (i.e., tricuspid) aortic valve. This approach has been implemented in this cohort by others ^1,2^⁠. Although some patients do present to the clinic with degenerative disease of a tricuspid aortic valve before the age of 65, extensive sensitivity analyses were previously performed by Saha et al. to validate this age cut-off to delineate BAV in UKB participants with cardiac MRI imaging available, in a similar study in UKB ^1^⁠. We did not repeat the extensive analyses of that previous work, but did carry out one sensitivity analysis using an age cut-off of 50 years, below which there would be very high confidence that a degenerate aortic valve would be developmentally abnormal (see Results). We specifically excluded participants with diagnoses of endocarditis at any time (an important cause of non-congenital aortic valve disease in younger patients). Participants with a code indicating presence of a heart defect of unconfirmed aetiology who did not meet the other inclusion requirements were excluded from both the case and control groups as their status could not be reliably assigned.

## Approach to distinguishing cases of ASD and PFO

Previous studies, conducted when only self-reported and HES-derived phenotypes were available in UKB, have used methods which exploit the increased risk of stroke due to ASD-induced atrial fibrillation as a means to correctly classify ambiguous ASD/PFO diagnoses ^1,3^. We used a different approach that took advantage of the recently available primary care data. Since additional PFO-specific codes are included in primary care data, participants with a potential ASD (Table S6E) were included in the case cohort only if their primary care records were available and they did not have a PFO-specific code (Table S6F). As primary care records are not available for all UKB participants, this will no doubt result in a failure to include some participants with ASDs as cases. However, we reasoned it was more important that we exclude PFOs from the case cohort, even at the cost of excluding some participants with genuine ASDs from the study. As with the unconfirmed CHD inclusion stream, participants with a code indicating presence of an ASD who did not meet all other inclusion requirements were assigned uncertain status and excluded from the control group. Finally, participants with codes for heart-related conditions which might lead to non-congenital heart defects were also excluded from the control group (Table S6G).

## Decision to exclude pulmonary TB diagnosis from main regression models

Pulmonary tuberculosis (TB) infection is a risk factor for COPD ^4^, however its role in COPD risk is more significant in countries where, unlike the UK, TB prevalence is high. Using EHR and self-reported illness data we identified only 489 cases of pulmonary TB in the entire study population, of which only 12 were CHD cases. These numbers are clearly too small for any useful conclusions to be drawn. To mitigate any risk that we were excluding an important risk factor, we performed a sensitivity analysis in which pulmonary TB diagnosis was included as a binary predictor in the fully adjusted Cox model from the main analysis (Table S8). This made a negligible difference to the HRs for CHD on COPD diagnosis, and all associations remained highly significant.

# References

1. Saha, P. *et al.* Substantial Cardiovascular Morbidity in Adults With Lower-Complexity Congenital Heart Disease. *Circulation* **139**, 1889–1899 (2019).

2. Helle, E. *et al.* Loss of function, missense, and intronic variants in NOTCH1 confer different risks for left ventricular outflow tract obstructive heart defects in two European cohorts. *Genetic Epidemiology* **43**, 215–226 (2019).

3. Williams, S. G. *et al.* Association of congenital cardiovascular malformation and neuropsychiatric phenotypes with 15q11.2 (BP1–BP2) deletion in the UK Biobank. *European Journal of Human Genetics* 1–9 (2020) doi:10.1038/s41431-020-0626-8.

4. Fan, H. *et al.* Pulmonary tuberculosis as a risk factor for chronic obstructive pulmonary disease: a systematic review and meta-analysis. *Annals of Translational Medicine* **9**, 390–390 (2021).

**Table S1**: Characteristics of the two main CHD subgroups. P-values refer to differences between participants of both sexes from each subgroup to the control group. Median/IQR for pack years of smoking were calculated using only values from ever-smokers. TDI – Townsend deprivation index.

|  | **Isolated AoV** | | **Noncomplex** | | **Control** | | **Isolated AoV** | **Noncomplex** |
| --- | --- | --- | --- | --- | --- | --- | --- | --- |
|  | **Male** | **Female** | **Male** | **Female** | **Male** | **Female** | **P-value** | |
| **n** | 1,253 (63.9) | 707 (36.1) | 626 (48.4) | 668 (51.6) | 216,791 (45.2) | 262,974 (54.8) | - | - |
| **Age, median (IQR)** | 60 (55, 64) | 59 (54, 63) | 59 (50, 64) | 57 (50, 63) | 58 (50, 63) | 57 (50, 63) | 2.06E-31 | 0.96 |
| **White, n (%)** | 1,215 (97.0) | 680 (96.2) | 585 (93.5) | 631 (94.5) | 205,288 (94.7) | 248,940 (94.7) | 3.33E-05 | 0.26 |
| **TDI, median (IQR)** | -1.99 (-3.56, 0.79) | -1.69 (-3.33, 1.16) | -1.61 (-3.3, 1.39) | -1.91 (-3.44, 0.8) | -2.15 (-3.66, 0.58) | -2.16 (-3.65, 0.44) | 6.60E-05 | 2.34E-05 |
| **BMI, median (IQR), kg/m2** | 28.1 (25.6, 31.5) | 27.9 (24.4, 32.1) | 27.6 (25.1, 30.3) | 26.7 (23.8, 30.5) | 27.3 (25.0, 30.0) | 26.1 (23.4, 29.7) | 1.98E-36 | 1.76E-03 |
| **Smoking status, n (%)** | | |  |  |  |  |  |  |
| **Current** | 158 (12.6) | 63 (8.9) | 70 (11.2) | 68 (10.2) | 27,154 (12.5) | 23,419 (8.9) | 0.29 | 0.89 |
| **Former** | 590 (47.1) | 260 (36.8) | 242 (38.7) | 209 (31.3) | 82,877 (38.2) | 82,535 (31.4) | 4.77E-16 | 0.79 |
| **Never** | 505 (40.3) | 384 (54.3) | 314 (50.2) | 391 (58.5) | 106,760 (49.2) | 157,020 (59.7) | 1.75E-17 | 0.74 |
| **Pack years of smoking, median (IQR)** | 25.9 (13.5, 41.0) | 21.5 (10.1, 36.0) | 24.0 (12.2, 38.6) | 18.4 (9.4, 30.9) | 21.0 (11.0, 35.0) | 16.5 (8.5, 28.1) | 6.71E-14 | 0.02 |
| **Other diagnoses, n (%)** | | |  |  |  |  |  |  |
| **Asthma** | 174 (13.9) | 141 (19.9) | 92 (14.7) | 123 (18.4) | 27,077 (12.5) | 37,328 (14.2) | 7.96E-04 | 1.08E-03 |
| **Hypertension** | 1,007 (80.4) | 518 (73.3) | 451 (72.0) | 389 (58.2) | 133,843 (61.7) | 128,858 (49.0) | 9.79E-100 | 1.58E-13 |

**Table S2**: Number at risk of COPD diagnosis in each group at five-year intervals, throughout the 20-year study period. Number at risk refers to the number of participants still alive at each time point; participants with prior COPD diagnoses are not excluded from these counts.

|  | **Study Year** | | | | |
| --- | --- | --- | --- | --- | --- |
| **No. at Risk** | **0** | **5** | **10** | **15** | **20** |
| **All CHD** | 3,385 | 3,385 | 3,385 | 3,296 | 3,095 |
| **Isolated AoV** | 1,960 | 1,960 | 1,960 | 1,901 | 1,776 |
| **Noncomplex** | 1,294 | 1,294 | 1,294 | 1,271 | 1,204 |
| **Control** | 479,765 | 479,765 | 479,758 | 475,372 | 464,394 |

**Table S3**: Hazard ratios (HR) for COPD diagnosis across CHD groups. This model is identical to the fully adjusted model presented in Table 2, with the exception that diagnosis of diabetes mellitus was included as a predictor in this model.

|  | **All CHD** | **Isolated AoV** | **Noncomplex** | **Control** |
| --- | --- | --- | --- | --- |
| **No. at Risk** | 3,385 | 1,960 | 1,294 | 479,765 |
| **COPD Diagnoses, n (%)** | 294 (8.7) | 176 (9.0) | 106 (8.2) | 14,854 (3.1) |
| **HR** | 2.18 | 2.1 | 2.35 |  |
| **95% CI** | (1.95, 2.45) | (1.81, 2.44) | (1.94, 2.84) |  |
| **P-Value** | 6.30E-40 | 1.69E-22 | 2.51E-18 |  |

**Table S4:** Effect of CHD on pre-bronchodilator FEV_1_/FVC ratio at recruitment, estimated using multivariable-adjusted linear regression. The fully adjusted model incorporates adjustment age at recruitment, sex, material deprivation (Townsend deprivation index), BMI and smoking status (current vs former vs never).

|  |  | **Full Cohort** | **Participants without pre-AC COPD/asthma diagnoses only** |
| --- | --- | --- | --- |
|  | **n (CHD cases)** | 268,549 (1,784) | 248,408 (1,568) |
| **Age/Sex Adjusted** | **Effect of CHD on FEV_1_/FVC, beta (95% CI)** | -0.006 (-0.008, -0.003) | -0.004 (-0.007, -0.002) |
|  | **P-Value** | 1.13e-04 | 5.41e-03 |
| **Fully Adjusted** | **Effect of CHD on FEV_1_/FVC, beta (95% CI)** | -0.006 (-0.009, -0.003) | -0.004 (-0.007, -0.002) |
|  | **P-Value** | 2.47e-05 | 2.18e-03 |

**Table S5**: Effect of CHD on an alternate, binary COPD outcome variable, defined based on the presence of a COPD diagnosis prior to recruitment and a measured pre-bronchodilator FEV_1_/FVC ratio <0.7 at recruitment. The fully adjusted model incorporates adjustment age at recruitment, sex, material deprivation (Townsend deprivation index), BMI and smoking status (current vs former vs never).

|  | **All CHD** | **Control** |
| --- | --- | --- |
| **No. at Risk** | 1,784 | 266,765 |
| **COPD Cases, n (%)** | 27 (1.5) | 1,751 (0.7) |
| **Age/Sex Adjusted:** | |  |
| **OR** | 2.05 | - |
| **95% CI** | (1.39, 3.01) | - |
| **P-Value** | 2.63E-04 | - |
| **Fully Adjusted:** | |  |
| **OR** | 1.81 | - |
| **95% CI** | (1.22, 2.68) | - |
| **P-Value** | 3.33E-03 | - |

**Table S6A**: Codes used in the identification of genetic syndromes and potentially confounding cardiac conditions. An excel version of this table is available at https://github.com/dombyrne/SciRep_Byrne_2022.

| **ICD-10** |
| --- |
| I231, I232, I510, I511, I512, I513, I514, I515, I516, Q90, Q900, Q901, Q902, Q909, Q91, Q910, Q911, Q912, Q913, Q914, Q915, Q916, Q917, Q92, Q920, Q921, Q922, Q925, Q926, Q927, Q928, Q929, Q93, Q930, Q931, Q932, Q933, Q934, Q935, Q937, Q938, Q939, Q96, Q960, Q961, Q962, Q963, Q964, Q968, Q969, Q874, Q875, Q878, Q851 |
| **ICD-9** |
| 7581, 7582, 75982, 7588, 7589, 7595, 7583, 7584, 7585, 7586, 7587, 7580 |
| **OPCS-4** |
| K556 |
| **CTV3** |
| G361., X200d, G5y9., XE2bE, G5y6., XE0VC, G5yy6, X202s, XaBtO, XaBtP, G5y0., XM1Ql, XaDyL, G5y1., X201X, X779A, Xa1dR, Gyu5g, XE0UX, G5y30, G5y31, G5y32, G5y33, G5y34, G5y35, X202t, X77zJ, XM1Qo, Xa3ki, PJ00., PJ01., PJ0.., X7694, X78Ek, X78El, XE1MZ, PJ20., PJ21., PJ2.., X78Em, X78En, PJ10., PJ11., PJ1.., X78Eo, X78Ep, PJ50w, X78Eq, X78Er, PJ50x, PJ510, PJ521, PJ522, PJ523, PJ524, X78Es, PJ500, PJ501, PJ502, PJ503, PJ504, PJ505, PJ506, PJ507, PJ508, PJ50y, PJ51., PJ51z, PJ52., PJ52z, PyuA0, X00kY, XaYQU, XaYQb, XaYQe, XaaLb, PJ50., PJ50z, X78Eh, XE2R2, PJ36., PJ370, PJ37z, X78Et, XE2R1, PJ38., XE1Ma, PJ32., PJ31., PJ33., PJ330, PJ33z, PKyz5, PyuA1, X78Al, X78Am, X78Eu, XE2ac, XE2ad, XSDcM, XaIwZ, XaYQ0, XaYQ1, XaYQ2, XaYYN, PJ35., XM1FU, PJ30., PJ3y., PyuA2, X00mm, X401C, XaB46, PJ3.., PJ3z., PJ631, PJ632, PJ633, PJ634, X78F1, Xa9D6, PJ635, PJ630, PJ636, PyuA5, X78Ey, X78F0, X78F2, XE1Me, PJ6.., PJ60., PJ62., PJ63., PJ63z, X401O, XE1Mf, 2G24., PKy2., X205W, XE2uf, Xa86f, PKy76, PKy77, PKy8., PKy8z, Pyu99, XaK9C, PGyy3, PKy1., PKy4., PKy58, PKy5A, PKy7B, PKy9., PKy90, PKy94, PKy95, PKy9z, Pyu9A, X00dy, X00dz, X00e1, X00kb, X00le, X101Z, X101a, X101b, X101c, X101d, X101e, X30LZ, X40JP, X40Wk, X50HS, X783S, X78VL, XE1Ml, Xa0ZQ, Xa0Zf, Xa1oH, XaDki, XaDkz, XaIyB, XaL2C, XaL2v, XaQke, XaX00, XaYQq, XaYQz, XaZWI, XaaJL, XabrJ, PK5.., X102q, X78E7, X78E8, PJy.., PJy0., PJy1., PJy10, PJy11, PJy12, PJy13, PJy1z, PJy2., PJyy0, PJyy1, PJyy2, PJyyz, PJyz., PyuAA, X20If, X78Eg, X78Ex, X78F3, X78F4, X78F5, X78F6, X78F7, X78F8, X78F9, X78FA, X78FB, X78FC, X78FD, XE1Mh, PJ..., PJ511, PJ520, PJ532, PJ533, PJz.., PJz0., PJz1., PJz2., PJz3., PJzz., X20Ig, X4019, X401A, X78Ei, X78Ej, X78E9, PJ34., X20IZ, X20Ia, X20Ib, X20Id, X78Ev, PJ4.., PJ530, PJ531, PyuA4, X78Ew, PJ5.., PJ534, PJ53z, XE1Mb, XE1Mc, XE1Md, PJ640, PJ64z, PJ6z., X78Ez, PJ7.., PJ70., PJ72., PJ73., PJ74., PJ7z., XE1Mg, XaMNd |

**Table S6B**: Codes used to identify heart defects of confirmed congenital aetiology. An excel version of this table is available at https://github.com/dombyrne/SciRep_Byrne_2022.

| **CHD Subtype** | **ICD-10** | **ICD-9** | **OPCS-4** | **CTV3** |
| --- | --- | --- | --- | --- |
| **AVSD** | Q212 | 74562, 74563 | K091, K092, K093, K095, K096, K098, K099 | 7906., 79060, 79061, 79062, 79063, 79064, 79065, 7906y, 7906z, P56.., P560., P56y., P56z., P56z1, P56z2, X00zM, X00zN, X00zO, X77tu, X77vj, X77wc, X77we, X77wf, X77wg, X77wh, X77wi, X77wj, X77wk, X77wl, X77wn, X77wo, X77wp, X77wq, X77wr, X77ws, X77wt, X77wu, X77wv, X77ww, X77wx, X77wy, X77wz, X77x0, X77x1, X77x2, X77x3, X77x4, X77x5, X77x6, X77x7, X77x8, X77x9, X77xA, X77xB, X77xC, X77xD, X77xE, X77xF |
| **Aortic atresia** | Q252 | 74720 |  | P7225, X780c, X781N, X77zr, X77zs, X77zu |
| **Aortic coarctation** | Q251 | 74721, 7471, 74710, 74711, 74719 |  | P71.., P710., P71z., P7222, X7803, X781C, X781E, X781F, X781G, X781I, X781K, X781L, X781M, XE1KN, XE2vv, XE2vw, X780b |
| **Aortopulmonary septum defect** | Q214 |  | L014 | 7A003, XE2uq, XaCGP, XaCGQ, XaCGR |
| **Atrial isomerism** | Q206, Q240, Q241 | 74680, 74681 |  | X77tl, X77v0, X77v1, X77v2, XC0E7 |
| **Congenital anomalies of the pericardium** |  | 74685 |  | X782L, X782M, P6yy4 |
| **Congenital aortic aneurysm** |  | 74727 |  | X780f, XE1KP, P72z1 |
| **Congenital aortic insufficiency** | Q231 | 7464 |  | P64.., P640., P641., P64z., X2018, X77zm, X77zw |
| **Congenital aortic stenosis** | Q253, Q230 | 7463, 74722 |  | P63.., P722., P7223, P7224, X777g, X77zn, X780Z, X780a, XE1KO |
| **Congenital coronary vessel anomalies** | Q245 |  |  | P6y4., P6y40, P6y41, P6y42, P6y43, P6y45, P6y46, P6y4z, P6yy0, P6yy1, P6yy6, X77uo, X77up, X77v8, X781v, X781w, X7822 |
| **Congenital mitral insufficiency** | Q233 | 7466 |  | P66.., X77xp |
| **Congenital mitral stenosis** | Q232 | 7465 |  | P65.., P650., P651., P652., P653., P65z., P6yyC, X77u5, X77vL, X77wB, X77xj, X77xk |
| **Congenital pulmonary stenosis/atresia** | Q220, Q221, Q243, Q255, Q256 | 74600, 74601, 74683, 74730, 74731, 74732 |  | P601., P601z, P602., P6020, P6021, P602z, P6y2., P732., P733., P735., P738., X77zT, X77zV, X77zX, X77zY, X77zZ, X77za, X77zc, X77zf, X77zg, X780C, X780E, X780F, X780G, XE1KQ |
| **Congenital pulmonary valve insufficiency** | Q222 | 74602 |  | P60z0, X201M, X77zR, X77zS |
| **Congenital subaortic stenosis** | Q244 |  |  |  |
| **Congenital tricuspid stenosis/atresia** | Q224, Q226 | 7461 |  | P61.., P610., P611., P61z., X201F, X201H, X77tX, X77u0, X77xH, X77xI, XE1KG |
| **Cor biloculare** |  | 7457 |  | P57.. |
| **Cor triatrium** | Q242 | 74682 | K203 | P6y1., X00z3, X77vK, XM1GB, XaLey |
| **DOLV** | Q202 |  | K084 | X010o, X010p, X010q, X010r, X77uH |
| **DORV** | Q201 |  | K081, K082, K083 | P511., P5110, P5113, P511z, X010l, X77uE, X77uF, X77uG, X77uI, XaLf6, XaLf7 |
| **Discordant atrioventricular connection** | Q205 |  |  | P512., X77to, X77tp, X77tr, X77tt, X77u2, X77u3, X77u4, X77u7, X77u8, X77u9, X77vr, X77vs, X77wH, X77wJ, X77xM, X77xP, X77xo, X77xr |
| **Double inlet ventricle** | Q204 |  |  | X77tw, X77tx, X77ty, X77tz, X77xG, X77xi |
| **Ebstein’s anomaly** | Q225 | 7462 |  | P62.., X77xT |
| **HLHS** | Q234 | 7467 | K175 | P67.., XaLfQ |
| **IAA** |  |  | L237 | P713., X780d, X781D, X781H, X781O, X781P, X781Q, XaBY6, XaLhA |
| **Other acyanotic congenital heart disease** |  |  |  | X77uX, X77v3, X77v4, X77v6, X77vA, X77vB, X77vC, X77vD, X77vM, X77vN, X77vO, X77yO, X77yP, X77yQ, X77yg, X77yi, XaLfX |
| **Other aortic valve anomaly** |  |  |  | X201B, X777f, X777h, X77zo, X77zp, X77zv, X77zy, X77zz, X7800, X7801 |
| **Other congenital aortic anomaly** |  |  |  | X7802, XE1KM, P7210, P7212, P7213, P7214, P7215, P7216, P7220, P7221 |
| **Other congenital malformations of aortic or mitral valves** | Q238, Q239 |  |  | Pyu24, Pyu2H, X2010, X777x, X777y, X7780, X77wD, X77wE, X77wF, X77wI, X77wL, X77wM, X77wN, X77wO, X77wQ, X77wS, X77wT, X77wU, X77wV, X77wW, X77wX, X77wY, X77wZ, X77wa, X77wb, X77xl, X77xm, X77xs, X77xt, X77xu, X77xv, X77xx, X77xz, X77y0, X77y3, X77y4, X77y5, X77y6, X77y7, X77y8, X77y9 |
| **Other congenital malformations of great veins** | Q268, Q269, Q260, Q261 | 74740, 74741 |  | P74z2, P74z3, X77uW, X77uZ, X77ub, X77uc, X77ud, X77ue, X77ug, X77uh, X77ui, Pyu29, X207c, XE1KT |
| **Other congenital tricuspid valve malformations** | Q228, Q229 |  |  | Pyu23, Pyu2G, X77vm, X77vn, X77vo, X77vt, X77vu, X77vv, X77vx, X77vz, X77w0, X77w1, X77w2, X77w3, X77w4, X77w5, X77w6, X77w7, X77w8, X77w9, X77xJ, X77xK, X77xN, X77xQ, X77xR, X77xS, X77xV, X77xW, X77xX, X77xY, X77xb, X77xc, X77xd, X77xe, X77xf, X77xg, X77xh |
| **Other cyanotic congenital heart disease** |  | 74684 |  | P60z1, P6yyA, P7217, P74.., P740., P7401, P740z, P74z0, P74z1, PK34., X77tZ, X77uQ, X77uR, X77uS, X77uT, X77uV, X77ua, X77uf, X77vF, X77vR, X77yG, X77yH, X77yI, X77yJ, X77yK, X77yW, X77yX, X77yZ, X77ya, X77yb, X77yc, X77yd, X77yj, X77yk, X77yl |
| **Other malformations of pulmonary valve or artery** | Q223, Q257 | 7460, 74608, 74609, 7473, 74733, 74734, 74738, 74739 | L101, L102, L103, L104, L108, L109, L121, L122, L123 | 7A08., 7A080, 7A082, 7A08y, 7A08z, 7A090, 7A091, 7A092, P60.., P600., P6010, P60z., P60z2, P60zz, P73.., P730., P731., P734., P73y., P73z., Pyu22, Pyu27, X77zU, X77zh, X77zi, X77zj, X77zk, X780B, X780D, X780H, X780I, X780J, X780K, X780P, X780Q, X780R, X780T, X780U, X780W, X781b, XE1KR, XE2Qj, Xa9Cm, XaCIC |
| **Other septal defects** | Q218, Q219 | 74560, 74561, 74568, 74569, 7458, 7459 | K121, K122, K123, K124, K125, K128, K129, K135, K138, K139, K148, K149, K158, K159, K168, K169 | 7909., 79090, 79091, 79092, 79093, 79094, 7909y, 7909z, 790A., 790Ay, 790Az, 790B., 790By, 790Bz, 790Cy, 790Cz, P5y.., P5z.., Pyu21, X77ta, X77tn, X77wm, XC0I6, XE0Ea, XE1KA, Xa3hW, XaLfD, XaLfI, XaLfJ, XaLfK |
| **PAPVR** | Q263, Q264 | 74743 | K202 | 790F1, P7400, P742., P74z7, P74z8, X77uj, X77uk, X77ul, X77um, X77un, XE1KS |
| **PDA** | Q250 | 7470 | L021, L022, L023, L024, L028, L029, L031 | 7A01., 7A010, 7A011, 7A012, 7A013, 7A01y, 7A01z, 7A020, P70.., X012J, X012K, X012L, X70Z2, X7815, X7816, X781R, X781U, X781W, X781Y, XE0M6 |
| **Single ventricle** |  | 7453 | K178, K179 | X77tv, XC0MC, P53.., X010P, X010Q, X010R, X010S, XaLfL, XaLfT, XaLfU |
| **TAPVR** | Q262 | 74742 | K071, K072, K073, K078, K079 | 7905., 79050, 79051, 79052, 7905y, 7905z, P741., P7410, P7411, P741z, P74z6 |
| **TGA** | Q203 | 74510, 74511, 74512, 74518, 74519 | K051, K052, K058, K059, K061, K062, K063, K064, K068, K069, K186 | 79030, 79031, 7903y, 7903z, 7904., 79040, 7904y, 7904z, P51.., P510., P5111, P5112, P51y., P51z., X010e, X010i, X010n, X77uA, X77uB, X77uC, XE0EY, XE1KB, XE1KC, XE2ur, Xa9Qh, XaLen, XaLeo, XaLep, XaLew, XaMKA, XaMKB, XaMKC, XaMKD, XaMqN |
| **TOF** | Q213 | 7452, 74520, 74521 | K041, K042, K043, K044, K045, K046, K048, K049, K183, K193, K242 | 7902., 79020, 79021, 79022, 79023, 7902y, 7902z, P52.., P520., P521., P52z., X010T, X010U, X010V, X010m, X77yB, X77yC, X77yD, X77yE, XE1KD, XaMK5, XaMK6, XaMK7 |
| **Truncus arteriosus** | Q200 | 7450 | K296, K297, L01, L011, L012, L013, L018, L019 | 7A00., 7A000, 7A001, 7A002, 7A00y, 7A00z, P500., P50z., X00xH, X00xK, X00xY, X012G, X012H, X77uJ, X77uL, X77uM, X77uN, X77zL, X77zM, X77zN, X77zO, X77zP, X77zQ, X7807, X7808, X7809, X780A, X780S, X780V, XE2Qh, XaC2P, XaMIC, X77z1 |
| **Unsp. Anomalies of great veins** |  | 7474, 74748, 74749 |  |  |
| **Unsp. acyanotic congenital heart disease** | Q248, Q249 | 74686, 74688, 74690, 74691, 74692, 74699 | K181, K185, K187, K188, K189, K191, K196, K198, K199 | 790D., 790D0, 790D4, 790Dy, 790Dz, 790E., 790E0, 790E5, 790Ey, 790Ez, P6..., P6y.., P6y3., P6y3z, P6y62, P6y63, P6y64, P6yy., P6yy2, P6yy3, P6yy5, P6yy7, P6yy9, P6yyB, P6yyD, P6yyz, P6z.., P6z0., P6z1., P6z11, P6z1z, P6z2., P6zz., Pyu25, X77tW, X77tY, X77th, X77tk, X77yA, X77yF, X77yY, X77zK, X780j, X780k, XC0Oc, XE1KH, XE1KJ, XM0v4, Xa6aC, XaLex, XaLgH, XaOiT |
| **Unsp. congenital aortic anomaly** | Q254 | 7472, 74723, 74724, 74725, 74726, 74728, 74729 | K374 | P72.., P720., P721., P721z, P72z., Pyu26, X778j, X7804, X780Y, X780i, X780l, X780m, X780n, X780o, X780q, X780r, X780s, X780t, X780v, X780w, X780x, X780z, X7817, X781h, XaDyY |
| **Unsp. congenital malformations of cardiac chambers** | Q208, Q209 |  | K088, K089 | P6y30, P6y6., P6y6z, X77tg, P6yy8, P6z10, Pyu20, Pyu2F, X77ti, X77tm, X77tq, XaLfB, XaLfC |
| **Unsp. cyanotic congenital heart disease** | Q258, Q259 | 74693 | K141, K142, K143, K144, K145, K151, K152, K161, K162, K163, K164, K171, K172, K173, K174, K176, K177, K182, K192, K241, L032, L051, L052, L053, L054, L058, L059, L061, L062, L063, L064, L065, L066, L067, L068, L069, L071, L072, L073, L074, L075, L078, L079, L081, L082, L083, L084, L086, L087, L088, L089, L091, L092, L098, L099 | XaLet, 790A0, 790A1, 790B0, 790C1, 790D1, 790D2, 790E1, 790E2, 7A03., 7A031, 7A032, 7A03y, 7A03z, 7A04., 7A040, 7A041, 7A042, 7A043, 7A044, 7A045, 7A04y, 7A04z, 7A05., 7A050, 7A051, 7A05y, 7A05z, 7A06., 7A060, 7A061, 7A062, 7A063, 7A06y, 7A06z, 7A07., 7A070, 7A071, 7A07y, 7A07z, P6z3., Pyu28, Pyu2J, X00yp, X00yq, X00yr, X00z4, X00z6, X00zD, X00zE, X00zF, X00zG, X00zH, X00zI, X00zJ, X00zK, X00zL, X00zk, X00zl, X00zm, X00zn, X00zo, X010A, X010B, X010C, X010Y, X010b, X010s, X010t, X010w, X010x, X010z, X0110, X0111, X0113, X0114, X0115, X011G, X011H, X011I, X011N, X011O, X011P, X011Q, X011R, X011S, X011T, X012N, X012O, X012k, X012l, X012m, X012n, X012o, X012p, X012q, X012r, X012s, X012t, X012u, X012z, X0130, X0131, X0133, X0134, X0135, X0136, X203H, X77yS, X77yT, X77yU, X77yV, X77ym, X780u, X780y, X780z, X7810, X781c, X781d, X781e, X90Q3, X90QY, XE0EZ, XE0Eb, XE0Ec, XE0Ev, XE0Ew, XE1KK, XE2tv, XM00Q, XM0C1, XM1IT, XSBNW, Xa3ki, XaLfM, XaLfN, XaLfO, XaLfP, XaLfS, XaLfW, XaLgn, XaLgo, XaLgp, XaLgq, XaLgr, XaMK8, XaMK9, XaMr9, XaMxn, XaPr5, XaPr7, XaPrD, XaPrE, XaXDa, P543., X010L, X010M, X010N |
| **VSD** | Q210 | 7454 | K111, K112, K113, K114, K115, K116, K117, K118, K119, K131, K132 | 7908., 79080, 79081, 79082, 79083, 79084, 7908y, 7908z, P54.., P540., P541., P544., P545., P54y., P54z., X010G, X010H, X010I, X010J, X010K, X010O, X70Z1, X77yn, X77yo, X77yp, X77yq, X77yr, X77ys, X77yt, X77yu, X77yv, X77yw, X77yx, X77yy, X77yz, X77z0, X77z2, X77z3, X77z4, X77z5, X77z6, X77z7, X77z8, X77z9, X77zA, X77zB, X77zC, XaLeq, XaLer, XaLfE, XaLfF, XaLqP |

**Table S6C**: Codes used to identify heart defects of unconfirmed congenital aetiology. An excel version of this table is available at https://github.com/dombyrne/SciRep_Byrne_2022. * - maximum age of 65 years at the time of this diagnosis code being present. ** - maximum age of 18 years at the time of this diagnosis code being present.

| **CHD Subtype** | **ICD-10** | **ICD-9** | **OPCS-4** | **Self-reported Illnesses** | **Self-reported Operations** | **CTV3** |
| --- | --- | --- | --- | --- | --- | --- |
| **Aortic insufficiency** | I351*, I352* |  |  | 1587* |  | G5410*, G5412*, X2017*, X2019*, X76KT*, X777a*, G5414* |
| **Aortic stenosis** | I350* |  | K312*, K322*, K352* | 1490* |  | G5411*, G5413*, X2011*, X2013*, X777W*, Xa0Ct*, 79161*, X00vQ*, 79171*, 79191*, X00vR*, X00vT*, X00vU* |
| **Aortic valve replacement** |  |  | K261*, K262*, K263*, K264*, K265*, K268*, K269*, K302* |  | 1099* | X00vS*, 79110*, 79111*, 79112*, XaBXo*, XaBXp*, XM1LC*, XM1LD*, XM1LE*, XM1LF*, XaQYo*, XaQYp*, 79113*, X00vV*, X00vW*, X00vi*, XM1LP*, 79114*, X00vF*, X00vJ*, X00vh*, 7911y*, 7911z*, X00v8*, X00vB*, X00vj*, X00vk*, X00vl*, XE0Ef*, 79151*, X00vZ*, X00va*, X00vf* |
| **Other aortic valve anomaly** | I35* | 4241* |  | 1586* |  | G541.*, G541z* |
| **Other malformations of pulmonary valve or artery** | I37 |  |  |  |  |  |
| **Pulmonary insufficiency** | I371, I372 |  |  |  |  | G5430, G5432, X201L, X201N, G5434 |
| **Pulmonary stenosis** | I370 |  |  |  |  | G5431, G5433, X201I, X778D, X778E |
| **Pulmonary valve defect or repair** |  | 4243 | K281, K282, K283, K284, K285, K288, K289, K304, K357, K314, K324, K354, K356 |  |  | G543., G543z, X0109, 79130, 79131, XaBXs, XaBXt, 79132, XM1LK, XM1LL, XM1LM, XM1LN, 79133, X00wD, 79134, X00vx, X00vy, X00vz, XaLgs, 7913y, 7913z, X00vq, X00vt, XE0Eh, 79153, X00wG, X00wH, X00wM, XaLfj, 79163, X00w5, 79173, 79193, X00w6, X00w7, X00w8, X00w9, XaLfi |
| **Subaortic stenosis** |  |  | K373 |  |  | P6y0.* |
| **Unsp. Heart surgery** |  |  |  |  | 1069** |  |
| **Unsp. acyanotic congenital heart disease** |  |  |  |  | 1097* |  |

**Table S6D**: Exclusionary codes for cases on unconfirmed CHD. An excel version of this table is available at https://github.com/dombyrne/SciRep_Byrne_2022.

| **ICD-10** |
| --- |
| I010, I011, I012, I018, I019, I050, I051, I052, I058, I059, I060, I061, I062, I068, I069, I070, I071, I072, I078, I079, I080, I081, I082, I083, I088, I089, I098, I090, I091, I092, I099, I342, I330, I339, I38, I390, I391, I393, I394, I398, B376, M321, I790, I791, M314, M315, M316, I270, I272, I231, I232 |
| **ICD-9** |
| 390, 3909, 391, 3910, 3911, 3912, 3918, 3919, 392, 3920, 3929, 393, 3939, 3941, 3950, 3951, 3952, 3971, 3979, 398, 3980, 3989, 3940, 3942, 3949, 932, 421, 4210, 4211, 4219, 4249, 931, 930, 4477, 4467, 4465, 4160 |
| **OPCS-4** |
| K311, K321, K351, K251, K252, K253, K254, K255, K258, K259, K301, K344 |
| **CTV3** |
| G010., XaDyK, G011., G012., G01y., G01y0, Gyu00, G01.., G01yz, G01z., G02z., XaDyJ, G110., X200r, X769E, X77xq, XE0UZ, Xa0Cy, G111., G112., Xa3fL, Gyu10, X7784, X7787, X7788, G11.., G11z., X200p, X7785, X7786, XE0UY, Xa3fK, Xa3fM, G120., G121., G122., Gyu11, G12.., G12z., Xa3fN, G1400, G1403, X201D, X77xO, G1401, G1404, X201G, X77wv, X77xL, XM00K, G1402, G1405, G140., Gyu12, X77vp, X77vw, X77xU, Xa6Zk, G140z, G542z, X201C, X76KU, X76KV, Xa3fO, G13.., G130., G131., G132., G133., G13y., G13z., G5441, G5440, G5442, Gyu13, G544., Gyu15, G141., G1410, G1411, G1412, G141z, Gyu14, X7782, X7783, G1y0., G14.., G14z., XE0Ua, Xa3fP, G10.., G100., G101., G102., G10z., G1..., G1y.., G1yz., G1yz0, G1yz1, G1yzz, G1z.., N0420, XE0W6, XaDyG, Xaagc, G113., G510., G5100, G5101, G5102, G510z, G511., G5115, G511z, X200h, X200j, X76G3, XE0Us, Xa7rt, Xa7rx, Xa7tF, Xa7tG, Xa7tH, G51.., G51z., G51z0, G51z1, G51z2, G51z3, G51z4, G51z5, G51zz, XE2QH, X705l, G755., G755z, Nyu41, X00Dy, X705m, G410., X2036, X2037, X2034, X2035, X2038, X2039, X203A, X203B, X203F, X203I, X203J, Xa0Cx, Xa0Cz, G361., X200d, G0..., G00.., G0y.., G0z.., M15y6, X701V, X701l, X7083, G020., G02.., G021., Xa0D0, X200s, AB414, AB4z3, AB4z4, G54z., G54z2, G54z3, G54z4, G54zz, X200f, X200k, X200l, XE0Uw, 79160, X00tg, X00uA, 79170, 79190, X00uB, X00uC, X00uD, 79100, 79101, XaBXl, XaBXm, 79102, X00uG, XE0Ee, XM1IU, XM1IV, XM1IW, XM1IX, 79103, X00uF, XM1LQ, 79104, 79180, X00tj, X00tk, X00tl, X00tx, X00ty, X00u1, X00u9, XaCG7, XaCG8, XaCI2, XaCI3, 7910y, 7910z, XE0Ed, 79150, X00uJ, X00uK, X00uP, 79183, X00u2, X00u3, X00un, X00uo, X00vK, X00vL, X00vu, X00vv, X00wo, X00wp, X00xN, X00xO, G767., Xa0kU, XaDyH, X200h, Xa7tF, G5100, G5114, G5112, G5113, G5101, X200j, G5115, G5110, A2704, X200m, X200i |

**Table S6E**: Codes used to identify potential ASD cases. An excel version of this table is available at https://github.com/dombyrne/SciRep_Byrne_2022.

| **ICD-10** |
| --- |
| Q211 |
| **ICD-9** |
| 7455, 74551, 74552, 74558, 74559 |
| **OPCS-4** |
| K10, K101, K102, K103, K104, K105, K108, K109, K094 |
| **CTV3** |
| P55.., P550., P552., P55y., P55z., X77up, X77uz, X77vY, X77vZ, X77va, X77vb, X77vc, X77vd, X77ve, X77vf, X77vg, X77vh, XE1KE, XE1KF, 79070, XaCGC, 79071, 79072, 79073, 79074, 7907y, X00yc, 7907., 7907z, X00z7, XaCHK, XaCHL, XaLes |

**Table S6F**: Codes used to identify cases of PFO. An excel version of this table is available at https://github.com/dombyrne/SciRep_Byrne_2022.

| **OPCS-4** |
| --- |
| K165 |
| **CTV3** |
| P551., X00z8, X00z9, XaCHN, XaCHO |

**Table S6G**: Codes used to identify potentially confounding cardiac conditions, identification of which resulted in exclusion from the control group. An excel version of this table is available at https://github.com/dombyrne/SciRep_Byrne_2022.

| **ICD-10** |
| --- |
| I010, I011, I012, I018, I019, I050, I051, I052, I058, I059, I060, I061, I062, I068, I069, I070, I071, I072, I078, I079, I080, I081, I082, I083, I088, I089, I098, I090, I091, I092, I099, I342, M321, I790, I791, M314, M315, M316, B376, I270, I272, I231, I232, I33, I330, I339, I38, I39, I390, I391, I393, I394, I398, Z941, I711, I712, I421, I422 |
| **ICD-9** |
| 390, 3909, 391, 3910, 3911, 3912, 3918, 3919, 392, 3920, 3929, 393, 3939, 3941, 3950, 3951, 3952, 3971, 3979, 398, 3980, 3989, 3940, 3942, 3949, 931, 930, 4477, 4467, 4465, 4160, 932, 421, 4210, 4211, 4219, 4249, 4411, 4412, 426, 4251, V421 |
| **OPCS-4** |
| K311, K321, K351, K251, K252, K253, K254, K255, K258, K259, K301, K344, L273, L288, K33, K331, K332, K333, K334, K335, K336, K338, K339, K011, K012, K018, K019, K021, K022, K023, K024, K025, K026, K028, K029, K245, K246, K247 |
| **CTV3** |
| G010., XaDyK, G011., G012., G01y., G01y0, Gyu00, G01.., G01yz, G01z., G02z., XaDyJ, G110., X200r, X769E, X77xq, XE0UZ, Xa0Cy, G111., G112., Xa3fL, Gyu10, X7784, X7787, X7788, G11.., G11z., X200p, X7785, X7786, XE0UY, Xa3fK, Xa3fM, G120., G121., G122., Gyu11, G12.., G12z., Xa3fN, G1400, G1403, X201D, X77xO, G1401, G1404, X201G, X77wv, X77xL, XM00K, G1402, G1405, G140., Gyu12, X77vp, X77vw, X77xU, Xa6Zk, G140z, G542z, X201C, X76KU, X76KV, Xa3fO, G13.., G130., G131., G132., G133., G13y., G13z., G5441, G5440, G5442, Gyu13, G544., Gyu15, G141., G1410, G1411, G1412, G141z, Gyu14, X7782, X7783, G1y0., G14.., G14z., XE0Ua, Xa3fP, G10.., G100., G101., G102., G10z., G1..., G1y.., G1yz., G1yz0, G1yz1, G1yzz, G1z.., N0420, XE0W6, XaDyG, Xaagc, G113., XE2QH, X705l, G755., G755z, Nyu41, X00Dy, X705m, G410., X2036, X2037, G41y0, X2033, X2034, X2035, X2038, X2039, X203A, X203B, X203F, X203G, X203I, X203J, Xa0Cx, Xa0Cz, G361., X200d, G510., G5100, G5101, G5102, G510z, G511., G5115, G511z, X200h, X200j, X76G3, XE0Us, Xa7rt, Xa7rx, Xa7tF, Xa7tG, Xa7tH, G51.., G51z., G51z0, G51z1, G51z2, G51z3, G51z4, G51z5, G51zz, 14S3., ZV421, G711., X204y, XaQZH, G712., X204L, X778h, G551., G5543, Gyu5M, X201Y, G0..., G00.., G0y.., G0z.., M15y6, X701V, X701l, X7083, G020., G02.., G021., Xa0D0, X200s, AB414, AB4z3, AB4z4, G54z., G54z0, G54z1, G54z2, G54z3, G54z4, G54zz, X200f, X200k, X200l, XE0Uw, X204c, X204d, X204e, X00y3, X200b, 79160, X00tg, X00uA, 79170, 79190, X00uB, X00uC, X00uD, 79100, 79101, XaBXl, XaBXm, 79102, X00uG, XE0Ee, XM1IU, XM1IV, XM1IW, XM1IX, 79103, X00uF, XM1LQ, 79104, 79180, X00tj, X00tk, X00tl, X00tx, X00ty, X00u1, X00u9, XaCG7, XaCG8, XaCI2, XaCI3, 7910y, 7910z, XE0Ed, 79150, X00uJ, X00uK, X00uP, 79183, X00u2, X00u3, X00un, X00uo, X00vK, X00vL, X00vu, X00vv, X00wo, X00wp, X00xN, X00xO, XaMmJ, XaLhL, XaLfr, XaLfs, XaLft, XaLfu, XaLfv, XaLfw, XaLfx, XaLfq, XaLfy, 79000, 79001, 7900y, 7900, 7900z, 79010, X00y4, X00y5, 79011, 79012, 79013, 79014, 79015, 7901y, 7901, 7901z, X00y1, XaLfa, XaMNL, X0105, G767., Xa0kU, XaDyH, X200h, Xa7tF, G5100, G5114, G5112, G5113, G5101, X200j, G5115, G5110, A2704, X200m, X200i |

**Table S6H**: Groupings of CHD diagnoses used to define each case subgroup. The isolated AoV defect group was limited to participants with an aortic valve defect only – participants with both an aortic valve defect and a noncomplex defect were placed in the noncomplex group. An excel version of this table is available at https://github.com/dombyrne/SciRep_Byrne_2022.

| **Noncomplex Defects** | **Complex Defects** | **Isolated AoV Defects** |
| --- | --- | --- |
| VSD | AVSD | Aortic insufficiency |
| ASD | Atrial isomerism | Aortic stenosis |
| PDA | Cor biloculare | Aortic valve replacement |
| Pulmonary stenosis | DOLV | Congenital aortic insufficiency |
| Pulmonary insufficiency | DORV | Congenital aortic stenosis |
| Aortic atresia | Discordant atrioventricular connection | Other aortic valve anomaly |
| Aortic coarctation | Double inlet ventricle |  |
| Aortopulmonary septum defect | HLHS |  |
| Congenital anomalies of the pericardium | IAA |  |
| Congenital aortic aneurysm | Other cyanotic congenital heart disease |  |
| Congenital coronary vessel anomalies | Single ventricle |  |
| Congenital mitral insufficiency | TAPVR |  |
| Congenital mitral stenosis | TGA |  |
| Congenital pulmonary stenosis/atresia | TOF |  |
| Congenital pulmonary valve insufficiency | Truncus arteriosus |  |
| Congenital subaortic stenosis | Unsp. congenital malformations of cardiac chambers |  |
| Congenital tricuspid stenosis/atresia | Unsp. cyanotic congenital heart disease |  |
| Cor triatrium |  |  |
| Ebstein’s anomaly |  |  |
| Other acyanotic congenital heart disease |  |  |
| Other congenital aortic anomaly |  |  |
| Other congenital malformations of aortic or mitral valves |  |  |
| Other congenital malformations of great veins |  |  |
| Other congenital tricuspid valve malformations |  |  |
| Other malformations of pulmonary valve or artery |  |  |
| Other septal defects |  |  |
| PAPVR |  |  |
| Unsp. acyanotic congenital heart disease |  |  |
| Unsp. Anomalies of great veins |  |  |
| Unsp. congenital aortic anomaly |  |  |
| Pulmonary valve defect or repair |  |  |
| Unsp. acyanotic congenital heart disease |  |  |
| Unsp. congenital aortic anomaly |  |  |

**Table S6I**: Numbers of participants in the final case cohort recorded as having each CHD subtype. An excel version of this table is available at https://github.com/dombyrne/SciRep_Byrne_2022.

| **CHD Diagnosis** | **N** |
| --- | --- |
| Aortic stenosis | 990 |
| Aortic insufficiency | 676 |
| Aortic valve replacement | 670 |
| Congenital aortic insufficiency | 348 |
| Unsp. acyanotic congenital heart disease | 303 |
| VSD | 186 |
| ASD | 186 |
| Unsp. Heart surgery | 185 |
| Other septal defects | 129 |
| Other aortic valve anomaly | 114 |
| Congenital aortic stenosis | 84 |
| PDA | 72 |
| Congenital pulmonary stenosis/atresia | 66 |
| Pulmonary insufficiency | 54 |
| Aortic coarctation | 45 |
| Congenital coronary vessel anomalies | 40 |
| Unsp. cyanotic congenital heart disease | 37 |
| Other malformations of pulmonary valve or artery | 35 |
| Unsp. congenital aortic anomaly | 33 |
| Pulmonary stenosis | 32 |
| TOF | 29 |
| AVSD | 28 |
| Atrial isomerism | 26 |
| PAPVR | 18 |
| Ebstein’s anomaly | 18 |
| Congenital mitral insufficiency | 17 |
| Other congenital malformations of aortic or mitral valves | 14 |
| Pulmonary valve defect or repair | 10 |
| Other congenital malformations of great veins | 8 |
| Congenital subaortic stenosis | 8 |
| Unsp. congenital malformations of cardiac chambers | 6 |
| TAPVR | 5 |
| Other congenital tricuspid valve malformations | 5 |
| TGA | 4 |
| DORV | 4 |
| Congenital pulmonary valve insufficiency | 3 |
| HLHS | 3 |
| Other congenital aortic anomaly | 3 |
| Congenital tricuspid stenosis/atresia | 3 |
| Aortopulmonary septum defect | 3 |
| Double inlet ventricle | 2 |
| DOLV | 2 |
| Truncus arteriosus | 2 |
| Subaortic stenosis | 2 |
| Congenital mitral stenosis | 2 |
| Discordant atrioventricular connection | 2 |
| Aortic atresia | 1 |
| IAA | 1 |
| Congenital aortic aneurysm | 1 |
| Congenital anomalies of the pericardium | 0 |
| Cor biloculare | 0 |
| Cor triatrium | 0 |
| Other acyanotic congenital heart disease | 0 |
| Other cyanotic congenital heart disease | 0 |
| Single ventricle | 0 |
| Unsp. Anomalies of great veins | 0 |
| Unsp. acyanotic congenital heart disease | 0 |

**Table S7A**: Codes used to identify cases of COPD. An excel version of this table is available at https://github.com/dombyrne/SciRep_Byrne_2022.

| **ICD-10** |
| --- |
| J41, J410, J411, J418, J42, J43, J431, J432, J438, J439, J44, J440, J441, J448, J449 |
| **ICD-9** |
| 491, 4910, 4911, 4912, 4918, 4919, 492 |
| **Self-reported Illnesses** |
| 1112, 1113 |
| **CTV3** |
| H31.., H310., H310z, H311., H311z, H3120, H3121, H313., H31y., H31z., H32.., H320., H3200, H3201, H3202, H3203, H320z, H321., H322., H32y., H32y0, H32y1, H32z. |

**Table S7B**: Codes used to identify cases of asthma. An excel version of this table is available at https://github.com/dombyrne/SciRep_Byrne_2022.

| **ICD-10** |
| --- |
| J45, J450, J451, J458, J459 |
| **ICD-9** |
| 493, 4930, 4931, 4939 |
| **Self-reported Illnesses** |
| 1111 |
| **CTV3** |
| H33.., H330., H3300, H3301, H330z, H331., H3310, H3311, H331z, H332., H33z., H33z0, H33z1, H33zz |

**Table S7C**: Codes used in the identification of pulmonary tuberculosis infections. An excel version of this table is available at https://github.com/dombyrne/SciRep_Byrne_2022.

| **ICD-10** |
| --- |
| A15, A150, A151, A152, A153, A154, A155, A156, A157, A158, A159, A16, A160, A161, A162, A163, A164, A165, A167, A168, A169 |
| **ICD-9** |
| 10, 100, 1000, 1001, 1002, 1003, 1004, 1005, 1009, 101, 1010, 1011, 1012, 1013, 1014, 1015, 1019, 108, 1080, 1081, 1082, 1083, 1084, 1085, 1089, 109, 1090, 1091, 1092, 1093, 1094, 1095, 1099, 11, 110, 1100, 1101, 1102, 1103, 1104, 1105, 1109, 111, 1110, 1111, 1112, 1113, 1114, 1115, 1119, 112, 1120, 1121, 1122, 1123, 1124, 1125, 1129, 113, 1130, 1131, 1132, 1133, 1134, 1135, 1139, 114, 1140, 1141, 1142, 1143, 1144, 1145, 1149, 115, 1150, 1151, 1152, 1153, 1154, 1155, 1159, 116, 1160, 1161, 1162, 1163, 1164, 1165, 1169, 117, 1170, 1171, 1172, 1173, 1174, 1175, 1179, 118, 1180, 1181, 1182, 1183, 1184, 1185, 1189, 119, 1190, 1191, 1192, 1193, 1194, 1195, 1199, 12, 120, 1200, 1201, 1202, 1203, 1204, 1205, 1209, 121, 1210, 1211, 1212, 1213, 1214, 1215, 1219, 122, 1220, 1221, 1222, 1223, 1224, 1225, 1229, 123, 1230, 1231, 1232, 1233, 1234, 1235, 1239, 128, 1280, 1281, 1282, 1283, 1284, 1285, 1289 |
| **OPCS-4** |
| E95, E958, E959 |
| **Self-reported Illnesses** |
| 1440 |
| **CTV3** |
| 1411, 65V9., 69A3., A101., A10y., A11.., A110., A111., A11y., A11z., A12.., A122., A1220, A1221, A122z, A124., A1247, A125., A1252, A12y., A12yz, A18.., A180., A1800, A1801, A18y., A18z., A1y.., AE0.., AE00., AE0z., Ayu10, Ayu11, Ayu12, Ayu13, Ayu14, Ayu17, Ayu18, Ayu19, AyuJ4, F4A55, H450., L173., L1730, L1732, L1733, L1734, L173z, Q4024, X70Gw, X70Gx, X70HC, X73T6, X73T7, X73T8, X73T9, X73TB, X73TC, X80aq, Xa5Vg, Xa5Vl, Xa5u9, Xa5uE, XaBE4, XaCL6, XaK3y, XaKB5, XaKB6, XaKB7, XaKB8, XaKB9, XaKBA, XaKBB, XaKBC, XaMGd, Xac8c, Xac8d, Xagd4, ZV011, ZV712, ZV741, x01J2, x01J3, x01J7 |

**Table S7D**: Codes used to identify cases of hypertension. An excel version of this table is available at https://github.com/dombyrne/SciRep_Byrne_2022.

| **ICD-10** |
| --- |
| I10, I11, I15 |
| **ICD-9** |
| 401, 4010, 4011, 4019, 402, 4020, 4021, 4029, 405, 4050, 4051, 4059 |
| **Self-reported Illnesses** |
| 1072 |
| **CTV3** |
| 6628, 662F., G2..., G20.., G200., G201., G202., G20z., G21.., G210., G210z, G211., G211z, G24.., G240., G240z, G241., G241z |

**Table S7E:** Codes used to identify cases of hypertension. An excel version of this table is available at https://github.com/dombyrne/SciRep_Byrne_2022.

| **ICD-10** |
| --- |
| E10, E11, E12, E13, E14 |
| **ICD-9** |
| 6840, V180, V771, 250 |
| **Self-reported Illnesses** |
| 1222, 1223 |
| **CTV3** |
| C11y0, 66A3., 66A4., 66A5., 66A8., 66A9., 66AR., C10.., C100., C1000, C1001, C100z, C101., C1010, C1011, C101y, C101z, C102., C1020, C1021, C102z, C103., C1030, C1031, C103y, C103z, C105., C1050, C1051, C105y, C105z, C106., C1060, C1061, C106y, C106z, C107., C1070, C1071, C1072, C107y, C107z, C1081, C1082, C1083, C1085, C1086, C1087, C1088, C1089, C108y, C108z, C1091, C1092, C1093, C1094, C1095, C1096, C1097, C10A0, C10A1, C10A3, C10A4, C10A5, C10A6, C10A7, C10B0, C10y., C10y0, C10y1, C10yy, C10yz, C10z., C10z0, C10z1, C10zy, C10zz |

**Table S8**: Hazard ratios (HR) for COPD diagnosis across CHD groups. This model is identical to the fully adjusted model presented in Table 2, with the exception that diagnosis of pulmonary TB was included as a predictor in this model.

|  | **All CHD** | **Isolated AoV** | **Noncomplex** | **Control** |
| --- | --- | --- | --- | --- |
| **No. at Risk** | 3,385 | 1,960 | 1,294 | 479,765 |
| **COPD Diagnoses, n (%)** | 294 (8.7) | 176 (9.0) | 106 (8.2) | 14,854 (3.1) |
| **HR** | 2.19 | 2.08 | 2.39 | - |
| **95% CI** | (1.95, 2.46) | (1.8, 2.42) | (1.97, 2.89) | - |
| **P-Value** | 3.96E-40 | 4.45E-22 | 4.31E-19 | - |
